# Double Trouble: A Case Report of Hydralazine-Induced Vasculitis and Lupus

**DOI:** 10.7759/cureus.83202

**Published:** 2025-04-29

**Authors:** Malika Ibrahim, Thanda Aung, Sravani Penumarty

**Affiliations:** 1 Department of Medicine, Division of Rheumatology, University of California Los Angeles David Geffen School of Medicine, Los Angeles, USA

**Keywords:** anca-associated vasculitis, hydralazine-induced diffuse alveolar hemorrhage, hydralazine-induced dual aav and dil, hydralazine-induced lupus syndrome, hydralazine-induced lupus syndrome (hils), hydralazine toxicity, systemic lupus erythematosus, systemic lupus erythromatosus

## Abstract

We present a rare case of hydralazine-induced antineutrophil cytoplasmic antibody (ANCA) vasculitis and lupus, characterized by the simultaneous and severe involvement of multiple organ systems. Following outpatient hydralazine dose escalation, a 77-year-old woman developed hypoxic respiratory failure secondary to diffuse alveolar hemorrhage, acute kidney injury with crescentic glomerulonephritis, and pancytopenia. Serologic testing revealed positive antinuclear antibody (ANA), dsDNA, anti-histone antibody, and P-ANCA, supporting the diagnosis of drug-induced autoimmune syndrome. This case highlights the risk for severe multi-organ dysfunction in drug-induced lupus and vasculitis, emphasizing the need for vigilance in recognizing such complications.

## Introduction

Antineutrophil cytoplasmic antibody (ANCA) vasculitis is a small vessel disease in which ANCAs, typically against myeloperoxidase or proteinase 3, activate neutrophils and cause rapidly progressive glomerulonephritis, pulmonary hemorrhage, and systemic inflammation [1]. Drug-induced lupus (DIL), often associated with hydralazine, is a reversible autoimmune syndrome marked by anti-nuclear and anti-histone antibodies and immune-complex deposition, producing lupus-like manifestations that resolve upon drug withdrawal [2]. Hydralazine-induced ANCA vasculitis and lupus represent a potentially life-threatening adverse drug reaction that requires prompt recognition and management. With an incidence of 5-10% in patients receiving hydralazine, particularly among slow acetylators, women, and those on higher doses or prolonged therapy [3,4], this condition involves drug-induced DNA demethylation leading to autoantibody formation against neutrophil components [5]. Unlike classic DIL, which typically presents with milder constitutional symptoms and arthralgias, hydralazine-induced ANCA vasculitis often manifests with severe multi-organ involvement including glomerulonephritis, alveolar hemorrhage, and systemic vasculitis [6,7]. Diagnosis relies on establishing a temporal relationship between drug exposure and symptom onset, positive serologic markers (ANA, anti-histone antibodies, ANCA), and exclusion of alternative etiologies [8]. This report highlights the case of a 77-year-old woman who developed acute kidney injury with crescentic glomerulonephritis, pancytopenia, and diffuse alveolar hemorrhage following hydralazine dose escalation in the outpatient setting during the months preceding hospitalization, emphasizing the need for heightened clinical vigilance for this severe complication in patients receiving hydralazine therapy.

## Case presentation

A 77-year-old woman with a significant medical history of thyroid cancer status post-thyroidectomy and breast cancer status post-mastectomy presented with acute renal failure and pancytopenia. A few weeks before her admission, she was experiencing severe fatigue, chills, poor appetite, and intermittent diarrhea. Her thyroid replacement therapy was uptitrated on an outpatient basis to alleviate some of her symptoms, but the adjustment proved unsuccessful.

On admission, she was afebrile with a blood pressure of 165/85 mmHg, a respiratory rate of 18, and was saturating 99% on room air. On examination, she had 4+ bilateral lower extremity edema extending to the knees. She had increased work of breathing but a normal cardiac exam. 

She had a long history of severe hypertension, managed with several antihypertensive medications, including bumetanide 1 mg daily, hydralazine 100 mg three times daily, and valsartan 160 mg daily. Upon evaluation, the patient was found to have an acute kidney injury. Her urinalysis showed proteinuria and hematuria in the sub-nephrotic range. Her admission labs also revealed pancytopenia and elevation in both her sedimentation rate and C-reactive protein (CRP). 

**Figure 1:**
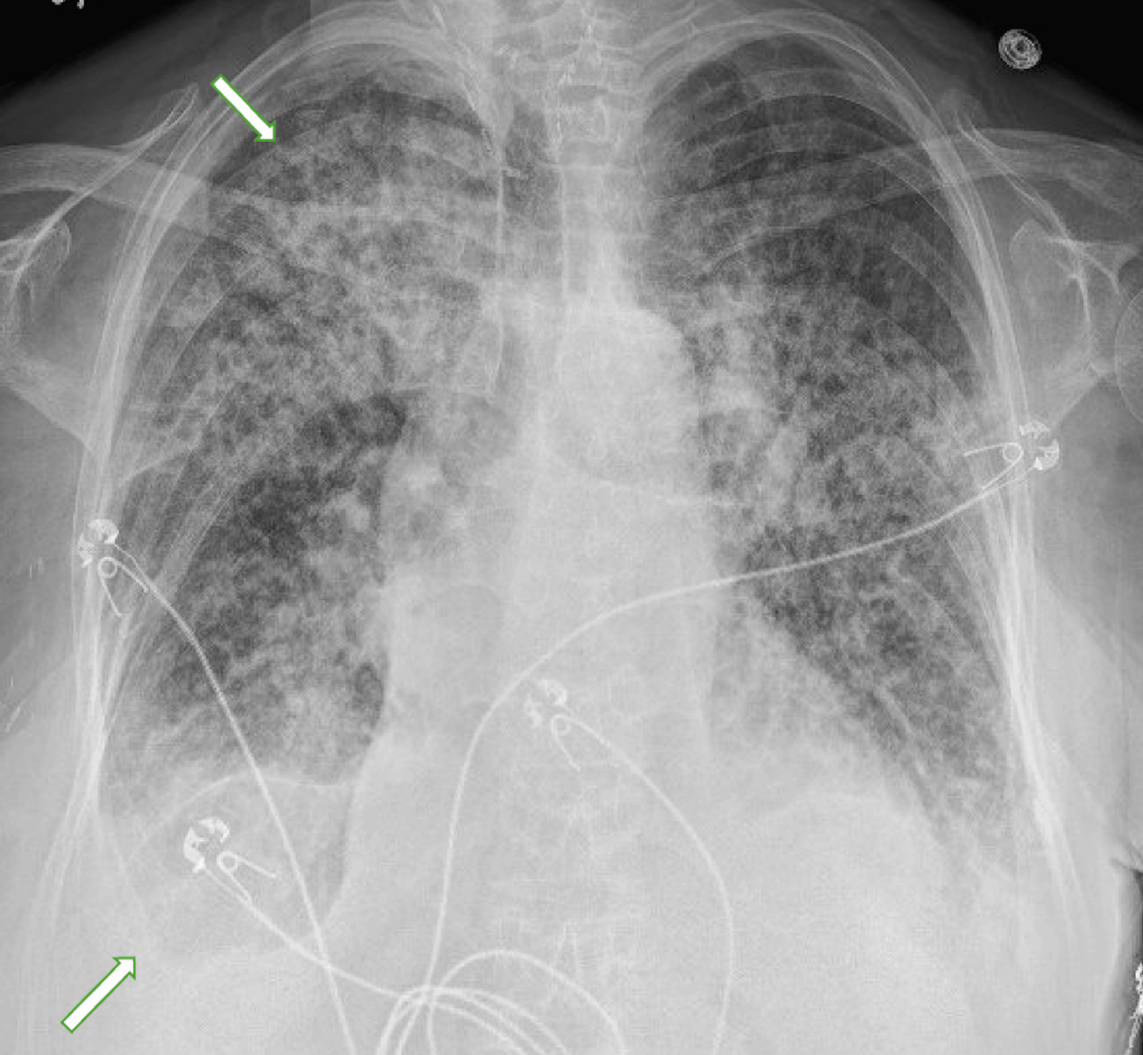
Chest X-ray showing a bilateral upper lobe greater than lower lobe airspace infiltrates and bilateral small pleural effusions

**Figure 2:**
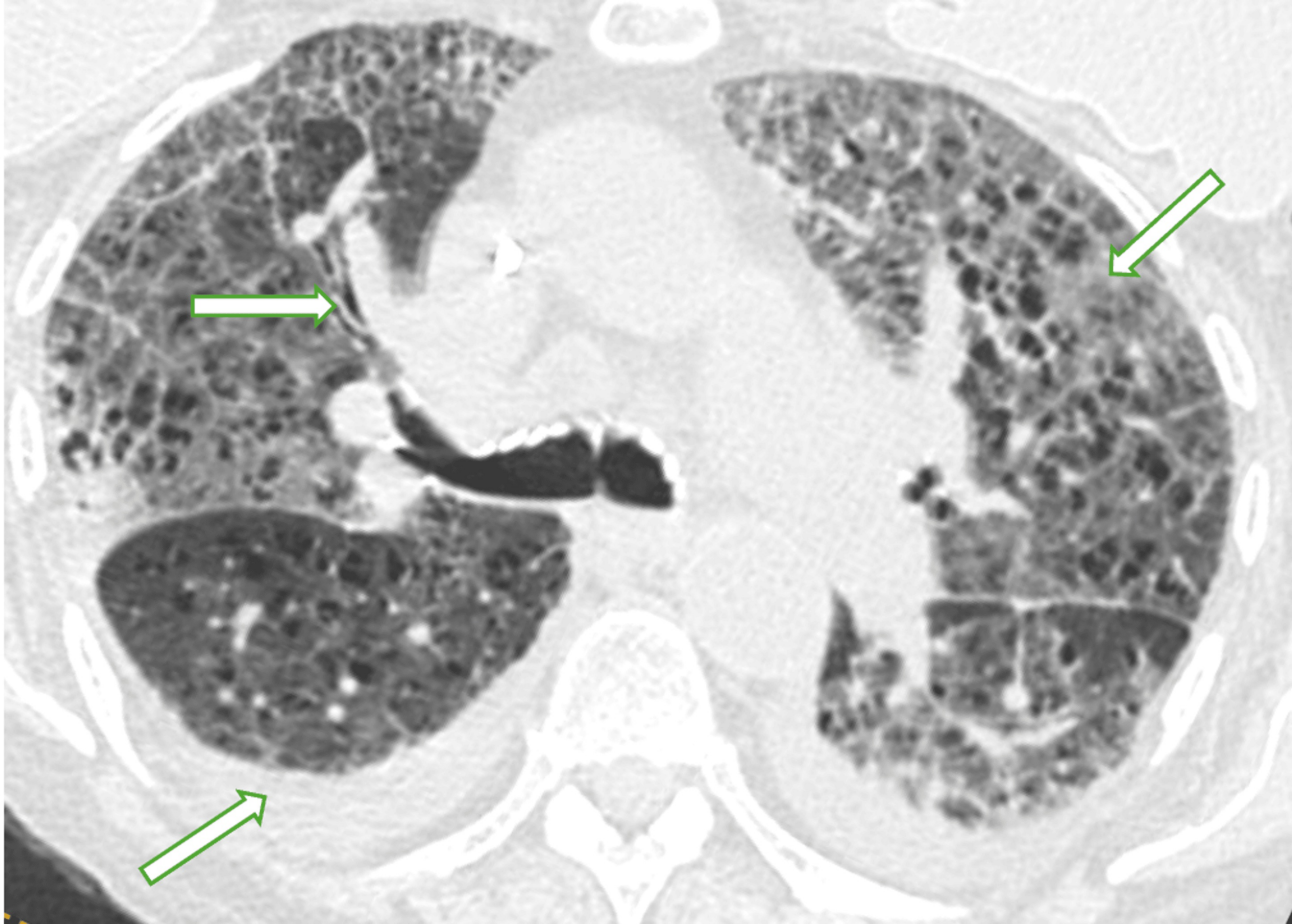
Follow-up CT chest without contrast showing interlobular septal thickening with patchy ground glass in the upper lobes, with a small right more than left pleural effusions with overlying atelectasis/pneumonia CT, Computed Tomography

In addition to her renal issues, she developed progressive anemia that remained unstable despite blood transfusions. On hospital day 3, she developed hypoxia requiring supplemental oxygen via a high-flow nasal cannula at 60L/min and 60% FiO2. A chest X-ray, shown in Figure 1, revealed diffuse airspace opacities, suggesting pulmonary edema or alveolar hemorrhage. The following day, her condition further deteriorated with the onset of frank hemoptysis and an increased need for oxygen, leading to her transfer to the intensive care unit (ICU). Due to her unstable vitals, bronchoscopy was deferred. A computed tomography (CT) scan of her chest, shown in Figure 2 completed five days into her ICU stay, displayed ground glass changes and pleural effusions with possible overlying atelectasis and or pneumonia. 

A series of autoimmune tests were ordered, detailed in Table 1, revealing low C3, low-normal C4, positive high-titer dsDNA and positive P-ANCA with titer elevation, raising concern for lupus nephritis versus ANCA-induced vasculitis. The differential diagnosis also included thrombotic thrombocytopenic purpura (TTP), hemolytic uremic syndrome (HUS), membranoproliferative glomerulonephritis, multiple myeloma, and cryoglobulinemia. 

**Table 1:** Laboratory results EGFR, Estimated Glomerular Filtration Rate; EIA, Enzyme Immunoassay; ANCA, Antineutrophil Cytoplasmic Antibodies; HPF, High-Power Field; CU, Chemiluminescent Units

Laboratory Test	Patient Value	Reference Range & Units
White Blood Cell Count	1.78 x10E3/uL	4.16-9.95 x10E3/uL
Hemoglobin	6.8 g/dl	11.6-15.2 g/dl
Hematocrit	22.1 %	34.9-45.2 %
Mean Corpuscular Volume	90.2 fL	79.3-98.6 fL
Mean Corpuscular Hemoglobin	29.4 pg	26.4-33.4 pg
Platelets	122 x10E3/uL	143-398 x10E3/uL
Absolute Reticulocyte Count	0.03 x10E3/uL	0.02-0.26 x10E3/uL
Red Blood Cell Morphology	Normal	Normal
Schistocytes	Negative	Negative
Haptoglobin	152 mg/dl	21-210 mg/dl
Lactate Dehydrogenase	286 U/L	125-256 U/L
Urea	51 mg/dl	7-22 mg/dl
Creatinine	5.3 mg/dl	0.6-1.3 mg/dl
eGFR	9 mL/min/1.73m2	>89 mL/min/1.73m2
Immunoglobulin G	1,181 mg/dl	70-1600 mg/dl
Immunoglobulin A	145 mg/dl	76-426 mg/dl
Immunoglobulin M	404 mg/dl	40-230 mg/dl
Complement 3	48 mg/dl	86-175 mg/dl
Complement 4	15 mg/dl	10-40 mg/dl
C-reactive Protein (CRP)	12.9 mg/dl	<0.8 mg/dl
Erythrocyte Sedimentation Rate (ESR)	39 mm/hr	<=25 mm/hr
Thyroid-Stimulating hormone (TSH)	11.9 mcIU/mL	0.03-4 mcIU/mL
Streptolysin O Antibody (ASO)	<55 IU/mL	<=330 IU/mL
Antinuclear Antibody	<1:40 titer	<1:40 titer
Double-Stranded DNA Antibody EIA	986 IU/mL	<=200 IU/mL
c-ANCA	1:40 titer	< 1:20 titer
P-ANCA	>-1:12850 titer	< 1:20 titer
Myeloperoxidase Antibody	128.4 CU	<20 CU
Proteinase-3 Antibody	69.9 CU	<20 CU
Histone Ab	5.3 Units	0.0-0.9 Units
Centrome B Antibody	<1.0 AI	<1.0 AI (Antibody Index)
PM/Scl 100 Antibody IgG	Negative	Negative
SM Antibody	<20 Units	<20 Units
RNP Antibody	<20 Units	<20 Units
SSA Antibody	<20 Units	<20 Units
SSB Antibody	<20 Units	<20 Units
SSA-52 Antibody (Ro52)	1 AU/mL	>=29 AU/mL
SSA-60 Antibody (Ro60)	0 AU/mL	>=29 AU/mL
Scl-70 Antibody	0 AU/mL	>=29 AU/mL
EJ Antibody	Negative	Negative
Ku Antibody	Negative	Negative
MDA5 Antibody	Negative	Negative
Mi-2 Antibody	Negative	Negative
NXP2 Antibody	Negative	Negative
OJ Antibody	Negative	Negative
PL-7 Antibody	Negative	Negative
PL-12 Antibody	Negative	Negative
P155/140 Antibody	Negative	Negative
SAE1 Antibody	Negative	Negative
SRP Antibody	Negative	Negative
TIF-1 Gamma Antibody	Negative	Negative
Jo-1 Antibody	Negative	Negative
HMGCR Antibody	<3 Units	0-19 Units
Thyroid Peroxidase Antibody	14 IU/mL	<=20 IU/mL
Thyroglobulin Antibody	<0.9 IU/mL	<=4 IU/mL
Cardiolipin Immunoglobulin A	<20 CU	<=20 CU
Cardiolipin Immunoglobulin G	<20 CU	<=20 CU
Cardiolipin Immunoglobulin M	167 CU	<=20 CU
Cryocrit	Negative	Negative
Urine Analysis
Protein	2+	Negative
Blood	3+	Negative
Red Blood Cell per HPF	>210 cells/HPF	0-2 cells/HPF
White Blood Cell per HPF	4 cells/HPF	0-4 cells/HPF
Hyaline Casts	>20/LPF	0-2/LPF

Further inquiry revealed that the patient had recently increased her hydralazine dosage to 100 mg three times daily approximately 1.5 months before this admission. This raised suspicion for hydralazine-induced lupus or a related vasculitis, further supported by a positive anti-histone antibody. 

On day 3 of hospitalization, she was treated with tranexamic acid for hemoptysis, high-dose steroids, a five-day course of plasmapheresis (PLEX), and a rituximab infusion as part of her ANCA-associated vasculitis vs DIL management. A hemodialysis catheter was inserted, and she underwent dialysis while being monitored for renal recovery. With the above treatment, the patient started showing clinical improvement. 

**Figure 3:**
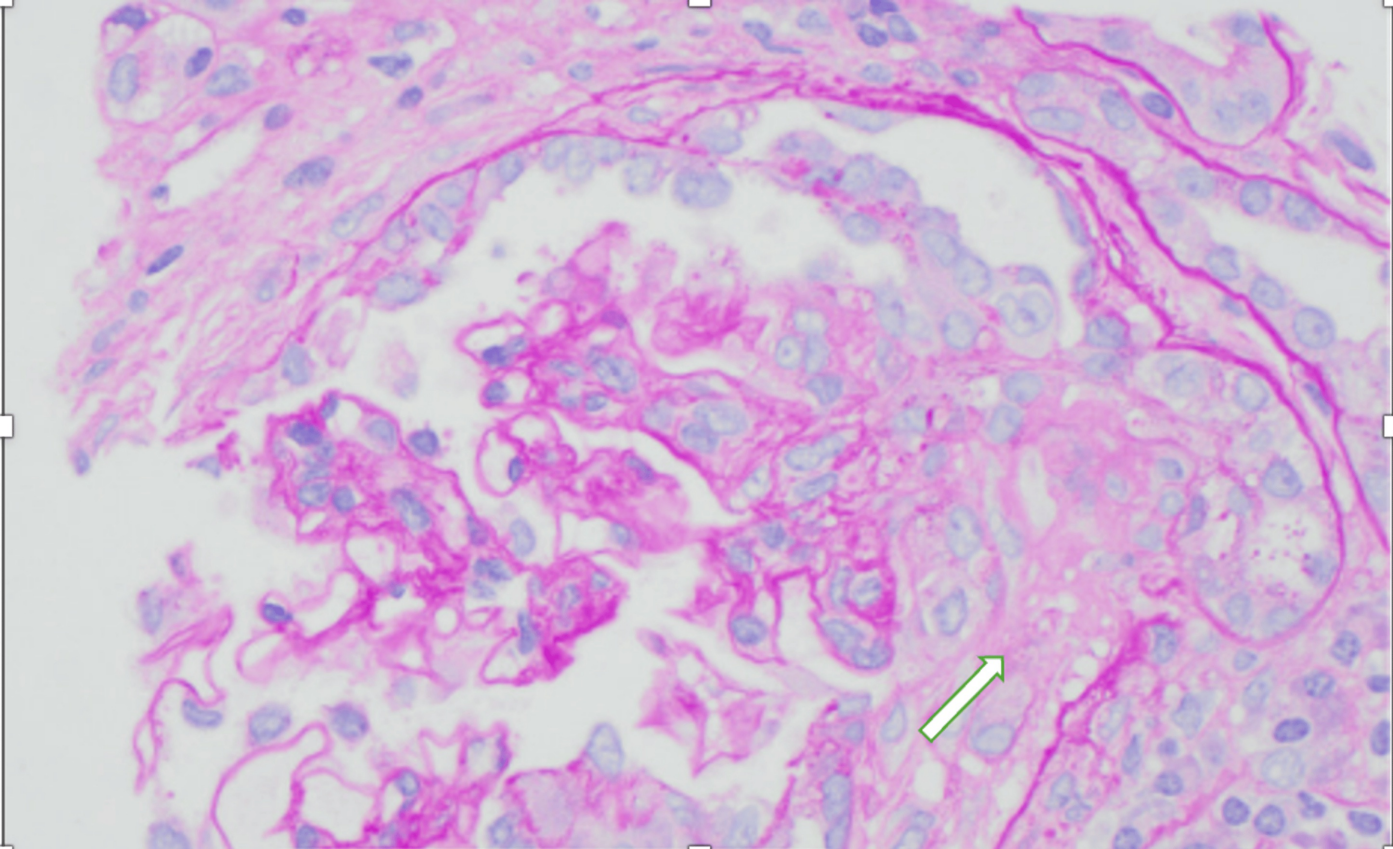
Kidney biopsy slide under light microscopy showing inflammatory cells and presence of fibrocellular crescents

**Figure 4:**
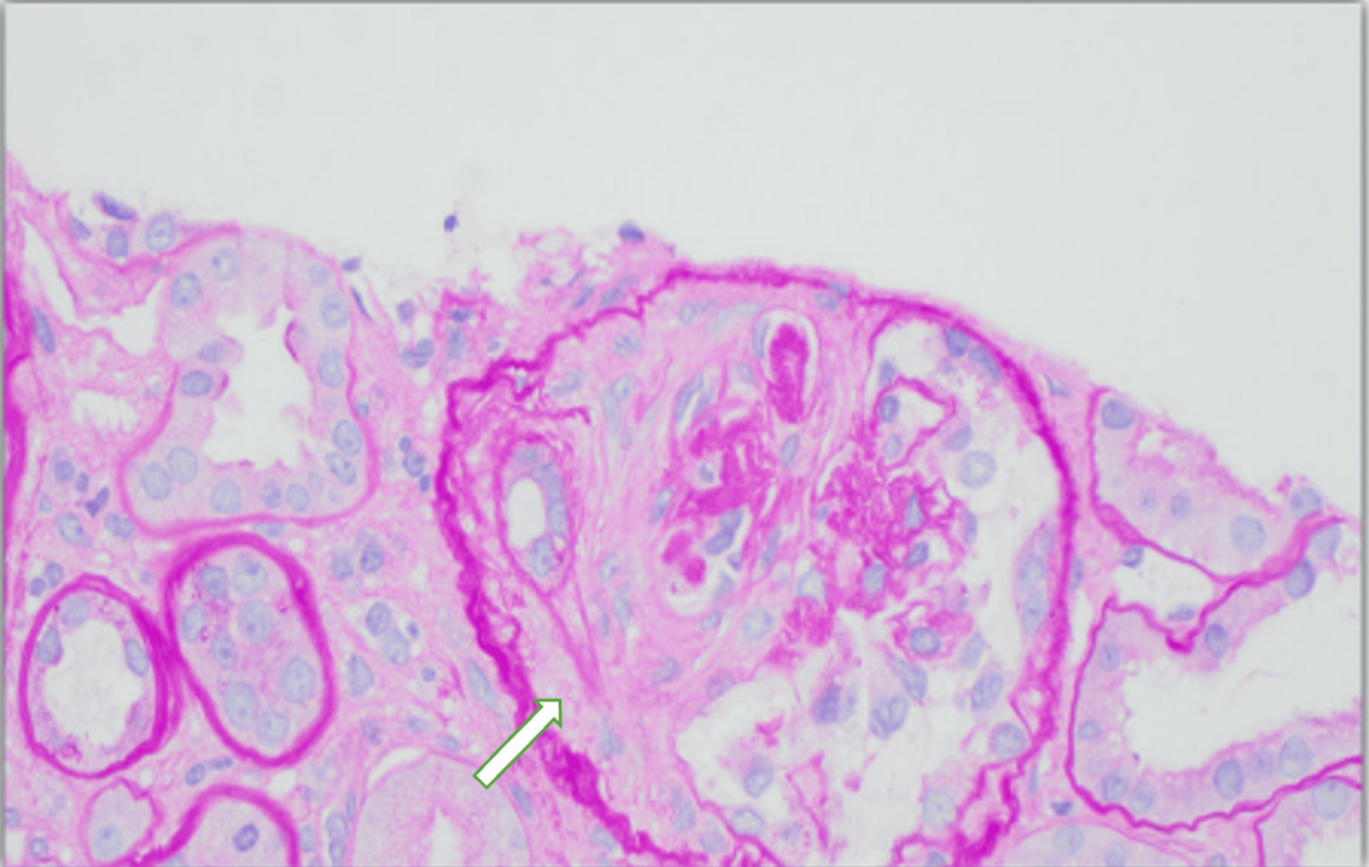
Kidney biopsy under light microscopy showing fibrous crescents

**Figure 5:**
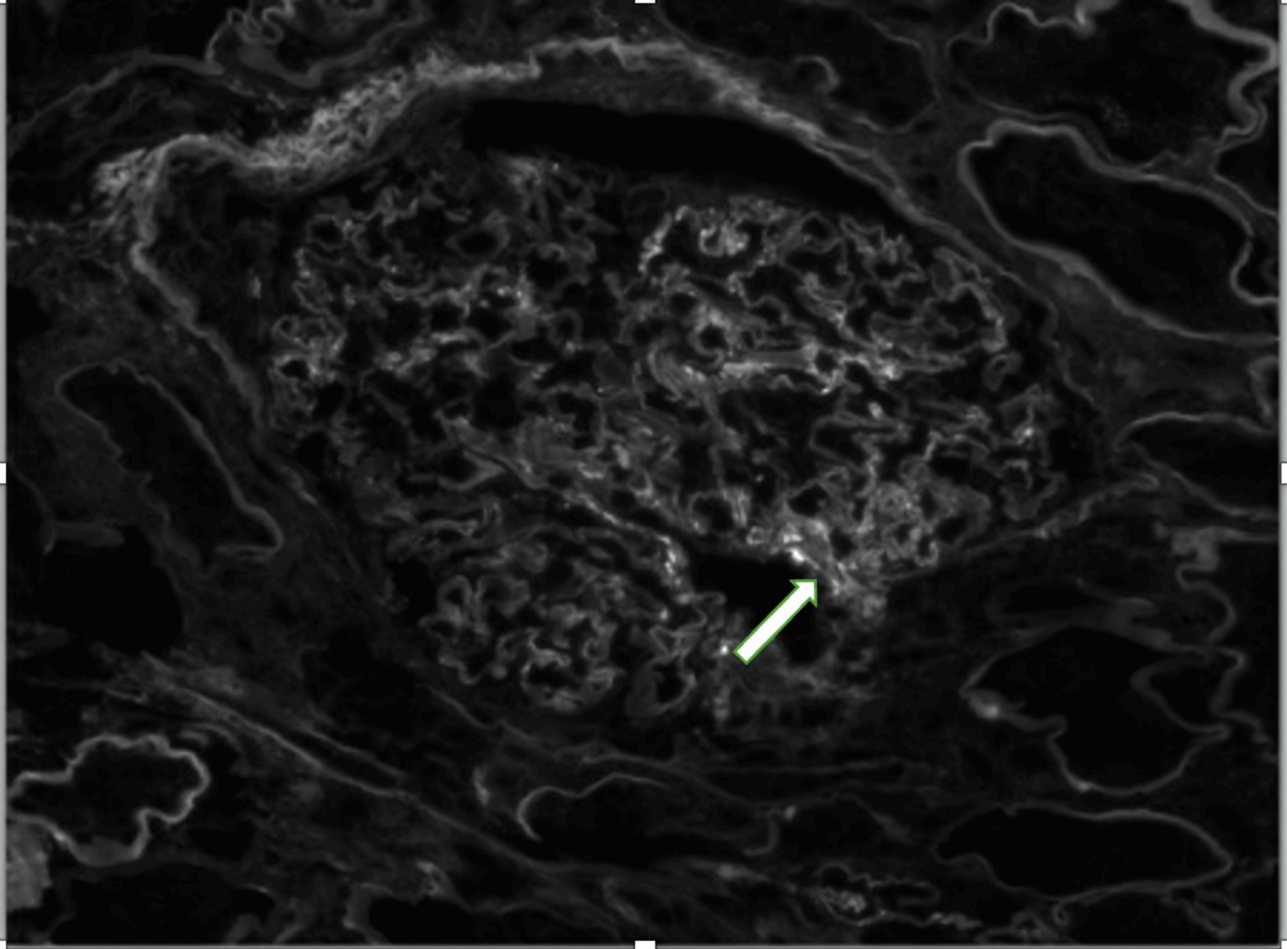
Kidney biopsy using immunofluorescence staining and microscopy showing segmental IgG deposition in the mesangial region

**Figure 6:**
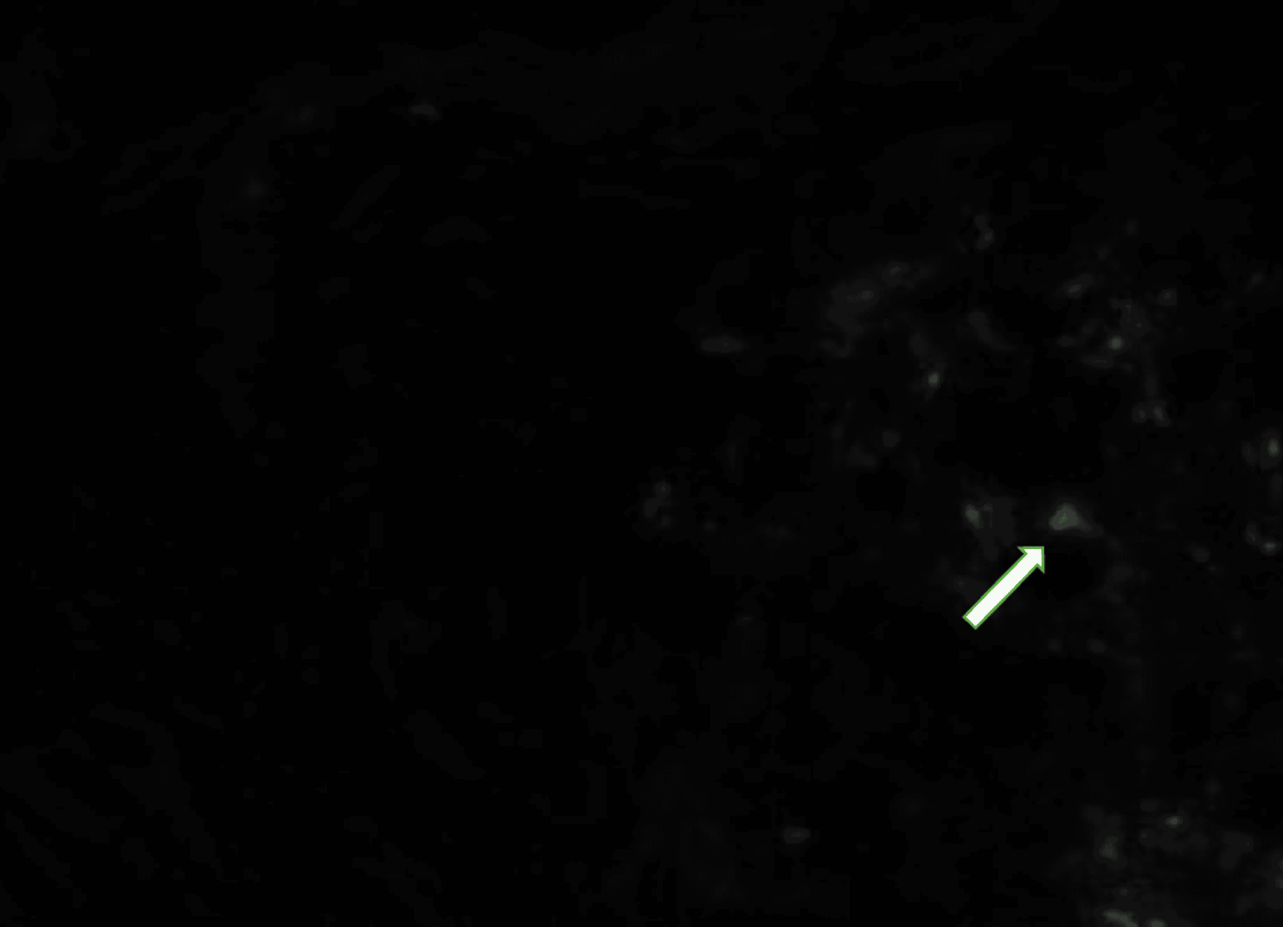
Kidney biopsy using immunofluorescence staining showing trace C3 deposition

A kidney biopsy, shown in Figures 3-6 with the corresponding pathology slides, was performed on hospital day 15 after the patient had stabilized. The findings were consistent with predominantly chronic crescentic glomerulonephritis. The biopsy revealed low-grade IgG/C3 deposits and mild tubulointerstitial scarring (25% involvement). Of the 18 glomeruli sampled, seven showed crescents (six fibrous, one fibrocellular). The low-grade deposits were mostly in mesangial regions but did not exhibit the characteristic "full house" pattern seen in lupus nephritis. These findings were compatible with a pauci-immune (ANCA-associated) process, which could be related to the noted hydralazine use. 

At the time of discharge, she was weaned off oxygen and scheduled for outpatient follow-up, including interval repeat imaging, pulmonary function tests, ongoing monitoring of renal improvement on dialysis, and rheumatology follow-up for immunosuppression management including steroid taper and rituximab infusions. 

## Discussion

This case illustrates the diagnostic and therapeutic challenges encountered in patients with multisystem involvement suggestive of an autoimmune process, particularly in the context of long-term medication use. The patient’s presentation, with acute kidney injury, pancytopenia, pulmonary hemorrhage, and elevated systemic inflammatory markers, prompted a broad differential that included lupus nephritis, ANCA-associated vasculitis, and other microangiopathic processes such as TTP or HUS. 

A critical aspect of this case was the history of hydralazine use, especially the recent up-titration to 100 mg three times daily. Hydralazine is a well-recognized trigger for DIL as well as for ANCA-associated vasculitis [9]. Factors leading to this spectrum of hydralazine-induced autoimmunity are not fully understood, but the current hypothesis includes that hydralazine alters neutrophil and lymphocyte function to promote the exposure of sequestered antigens and decrease central tolerance. This, in turn, leads to ANCAs and/or anti-nuclear antibodies (ANA), which may lead to overt systemic autoimmunity [10]. Notably, hydralazine-associated organ damage can be extensive, and in cases of hydralazine-induced ANCA vasculitis, the kidneys are frequently affected, manifesting as rapidly progressive glomerulonephritis with crescent formation, as seen in our patient. Such renal involvement can lead to irreversible kidney injury if not promptly identified and managed [6]. Additionally, though rare, pulmonary involvement evidenced by diffuse alveolar hemorrhage and interstitial changes on imaging in our case can occur leading to life-threatening respiratory compromise [11,12]. Lionaki et al. described an increased risk for developing hydralazine-induced ANCA-associated vasculitis with a longer duration and higher doses of hydralazine therapy especially in slow acetylators, female patients, and patients with a history of thyroid disease, a phenotype relevant to our case who suffered from hypothyroidism with futile up-titration of thyroid replacement therapy in the period leading to hospitalization [13]. 

Previous case reports reveal that most patients with drug-induced ANCA-associated vasculitis have MPO-ANCA, frequently in very high titers, as found in our patient [5]. However, the complete serologic profile of this patient further confounded the diagnostic picture. Although hydralazine-induced lupus is classically characterized by positive anti-histone antibodies with an absence of anti-dsDNA antibodies [14], our patient demonstrated elevated dsDNA titers. The coexistence of anti-dsDNA, typically seen in idiopathic SLE, with high-titer anti-histone antibodies, low C3 levels, pancytopenia, and constitutional symptoms complicates the clinical assessment, suggesting an overlap syndrome and underscoring the continuum between DIL and ANCA-associated vasculitis [6]. In experimental evidence, data demonstrated that patients treated with hydralazine recognize a broader array of auto-antigenic epitopes, which may explain the spectrum of antibody serology in our case [15]. 

Although DIL remained a consideration based on serology, the kidney biopsy findings of predominantly chronic crescents, low-grade IgG and C3 immune deposits, and the absence of a “full house” immunofluorescence pattern strongly favored a pauci immune process over classic lupus nephritis. These histopathologic features are consistent with ANCA-associated vasculitis and, in the context of hydralazine exposure, support a diagnosis of hydralazine-induced ANCA vasculitis. 

Management of this patient was complex and necessitated rapid intervention due to the progression of both renal and pulmonary involvement, and prompt discontinuation of the offending agent, hydralazine. The initiation of pulse-dose steroids, plasmapheresis, and rituximab reflects the accepted aggressive approach aimed at controlling the autoimmune process and minimizing irreversible organ damage in such cases [16]. This case underscores the importance of recognizing the full spectrum of hydralazine-induced organ injury. Early recognition of both hydralazine-associated ANCA vasculitis and DIL is crucial, as the extent of organ damage, ranging from severe renal impairment to life-threatening pulmonary hemorrhage, demands prompt and targeted therapy to improve patient outcomes. 

## Conclusions

This case illustrates the potentially life-threatening nature of hydralazine-induced ANCA vasculitis and lupus, characterized by multi-organ involvement including pulmonary hemorrhage, crescentic glomerulonephritis, and pancytopenia. The temporal relationship between hydralazine dose escalation and symptom onset, coupled with positive serologic markers, underscores the importance of medication review in patients presenting with unexplained multi-system disease. Clinicians should maintain a high index of suspicion for drug-induced autoimmunity, particularly in elderly patients and those with risk factors such as higher doses or prolonged therapy. Early recognition, prompt discontinuation of the offending agent, and appropriate immunosuppressive therapy are crucial for preventing irreversible organ damage. 
